# Meaningful digital mobility outcomes for people with Parkinson's disease: Insights grounded in diverse lived experience

**DOI:** 10.1177/1877718X261494917

**Published:** 2026-10-09

**Authors:** Jack Lumsdon, Ríona Mc Ardle, Megan Hanrahan, Alison Keogh, Cameron Wilson, Tova Gur Arieh, Alastair J Noyce, Essa Bhadra, Johanna Graeber, Lova Hulst, Moran Gilat, Heiko Gassner, Jürgen Winkler, Walter Maetzler, Anat Mirelman, Alison J Yarnall, Lynn Rochester, Katie Brittain

**Affiliations:** 1 Population Health Sciences Institute, Faculty of Medical Sciences, 5994 Newcastle University, Newcastle Upon Tyne, UK; 2 NIHR Newcastle Biomedical Research Centre, Newcastle University, The Newcastle upon Tyne Hospitals NHS Foundation Trust, Newcastle upon Tyne, UK; 3 Translational and Clinical Research Institute, Faculty of Medical Sciences, Newcastle University, Newcastle Upon Tyne, UK; 4 Insight for Data Analytics, 8797 University College Dublin, Dublin, Ireland; 5 School of Medicine, 8809 Trinity College Dublin, Dublin, Ireland; 6 Department of Public Health and Primary Care, School of Clinical Medicine, 2152 University of Cambridge, Cambridge, England; 7 Mobilise-D Patient and Public Advisory Group, Newcastle upon Tyne, UK; 8 Wolfson Institute of Population Health, 4617 Centre for Preventive Neurology, Queen Mary University of London, London, UK; 9 Department of Neurology, The Royal London Hospital, Barts Health NHS Trust, London, UK; 10 Department of Neurology, 9179 University Hospital Schleswig-Holstein, Kiel University, Kiel, Germany; 11 Department of Rehabilitation Sciences, 26657 Research Group for Neurorehabilitation (eNRGy), KU Leuven, Leuven, Belgium; 12 Department of Molecular Neurology, 27168 University Hospital Erlangen, Erlangen, Germany; 13 28450 Fraunhofer Institute for Integrated Circuits IIS, Erlangen, Germany; 14 Laboratory for Early Markers of Neurodegeneration, Center for the study of Movement, Cognition and Mobility, Sagol School of Neuroscience, Faculty of Medicine and Health Sciences, 26738 Neurological Institute, Tel Aviv Medical Center, Tel Aviv University, Tel Aviv, Israel; 15 5983 Newcastle upon Tyne Hospitals NHS Foundations Trust, Newcastle upon Tyne, UK

**Keywords:** Parkinson disease, gait, mobility limitation, digital health, digital technology, diversity, equity and inclusion, clinical trials

## Abstract

**Background:**

Digital mobility outcomes (DMOs) have been cited as potential clinical endpoints for trials in Parkinson's disease (PD). Current evidence from the Mobilise-D consortium suggests that selected DMOs are technically valid, with emerging research on their clinical validity. However, regulatory qualification of DMOs requires additional evidence of their meaningfulness to people with PD.

**Objective:**

To identify meaningful DMOs by understanding how mobility symptoms impact aspects of health that matter to people with PD.

**Methods:**

Purposive sampling recruited a diverse sample of people with PD to participate in semi-structured interviews. Content analysis identified frequent and bothersome mobility symptoms and their impacts on meaningful aspects of health. Twelve experts mapped meaningful symptoms to relevant DMOs through a nominal group consensus.

**Results:**

Seventy-two people with PD participated, inclusive of under-served groups: ethnic minorities (14%) or LGBTQIA + backgrounds (14%), in older age (3%) or with young-onset PD (13%), living rurally (31%) or in a deprived area (19%). Shuffling gait, bradykinesia and balance issues were the most frequent symptoms, and the most bothersome and impactful on meaningful aspects of health (i.e., activities of daily living, life-space mobility, emotional wellbeing) among participants who experienced them. Less frequent, but still highly bothersome and impactful symptoms included freezing of gait, fatigue and weakness. Expert consensus suggested that walking speed, stride length and turn duration were the most relevant DMOs to reflect these meaningful symptoms.

**Conclusion:**

Findings offer an inclusive, meaningful framework for digital assessment of mobility in PD, grounded in lived experience and consistent with regulatory guidance.

## Plain Language Summary

Making digital mobility measures meaningful for people with Parkinson's disease: Insights from diverse groups

Problems with mobility, especially walking, are some of the most challenging symptoms for people with Parkinson's disease. To develop better treatments, researchers need to measure changes in mobility in ways that are both accurate and meaningful to those living with the condition. Wearable digital devices offer a way to track movement continuously in everyday life, but it is not always clear which measures matter most to patients.

In this study, we spoke with 72 people with Parkinson's disease from a wide range of backgrounds, including people from ethnic minority and LGBTQIA + communities, those aged over 80 years, people with young-onset Parkinson's disease, and those living in rural or lower-income settings. We asked about their experiences of mobility symptoms and how these affected their daily lives, independence, and wellbeing.

Participants told us that shuffling steps, slow movement (bradykinesia), and balance issues were the most important symptoms. These were not only common, but also had the greatest impact on everyday activities, the ability to get around, and emotional wellbeing. Other important symptoms were freezing of gait, fatigue and weakness. We then worked with an additional 18 people with Parkinson's disease and three carers through public patient involvement workshops, to check our findings reflected real experiences.

Next, a group of technical and clinical expert practitioners in Parkinson's disease and digital health linked these key symptoms to specific digital measures of mobility. They agreed that walking speed, stride length, and turning time are the most relevant ways to capture these meaningful symptoms using wearable devices.

These findings will help researchers choose better digital measures in future studies, particularly when testing treatments aimed at slowing the progression of Parkinson's disease.

## Introduction

Parkinson's disease (PD) is a progressive neurodegenerative condition, affecting an estimated 11.77 million people worldwide.^1^ It is characterised by cardinal motor symptoms – bradykinesia, rigidity, tremor and postural instability – along with gait and balance impairments and a wide range of non-motor features.^2–4^ In the absence of a cure, PD treatment and management predominately focuses on alleviating motor symptoms and supporting physical mobility. The development of new pharmacological treatments are progressing, but these advances depend on sensitive, patient-centric endpoints that can capture therapeutic effects more accurately and sensitively than traditional clinical assessments, such as the Movement Disorders Society Unified Parkinson's Disease Rating Scale.^5^ Biomarker-based endpoints, such as neuroimaging methods and quantitative seed amplification assays to detect pathological alpha synuclein have been included in PD clinical trials; however, these approaches primarily provide biological or mechanistic information at discrete time points and may not capture aspects of disease that are directly meaningful to patients.^6^ Digital endpoints are gaining considerable traction as they can enable continuous remote monitoring of PD symptoms in the real world, supporting objective, ecologically valid data collection with the potential to facilitate decentralized clinical trial designs.^7^ Amongst these, digital mobility outcomes (DMOs) have emerged as particularly promising candidate endpoints.^8–10^ DMOs are outcomes derived from digital assessment of walking and related movements, typically using wearable technologies such as accelerometers and inertial measurement units. A recent systematic review reported DMOs as the most commonly employed digital outcomes in pharmaceutical trials for PD, highlighting both their perceived clinical relevance and their potential utility as trial endpoints.^11^

Physical mobility is defined as the ability to move freely and easily without a vehicle.^12^ DMOs can capture and quantify multiple aspects of physical mobility performance, including the quantity (e.g., amount of walking, step count) and quality of walking (e.g., walking speed, stride length).^8,13^ DMOs can capture real-world walking which provides a more ecologically-valid indication of function compared to traditional clinical snapshots.^10^ Real-world DMOs are particularly relevant to PD, with emerging evidence indicating that they might help identify individuals at risk of developing the condition,^14,15^ detect subtle motor changes in the prodromal and early stages,^16,17^ sensitively track disease progression over time,^18^ and act as an objective outcome measure to evaluate the efficacy of pharmacological therapies.^9,19^

Qualification of clinical endpoints for regulatory approval requires a robust evidence base to show that the outcomes are both technically and clinically valid, sensitive to clinically meaningful change, clearly set out a context of use and measure discrete outcomes which are meaningful to the target population.^20–23^ The Mobilise-D consortium has made significant strides to qualify DMOs derived from a lumbar-based inertial measurement unit as clinical endpoints in a range of health conditions, including PD.^8,12^ To date, they have demonstrated technical validity of 24 DMOs for use in PD, with further work ongoing to determine clinical validity, clinically meaningful changes and the context of use.^9,13,24^ However, a gap remains regarding the establishment of “meaningfulness” of DMOs for people with PD within the context of clinical trials, a critical requirement for regulatory qualification to ensure treatments positively impact what matters most to patients.^12,25^

A “meaningful” outcome is defined as one that is associated with either symptoms or functional impacts which are prevalent and personally bothersome to the patient.^26^ In PD, motor symptoms related to mobility (e.g., shuffling gait) are both prevalent and bothersome, consistently affecting people with PD across the disease course and limiting their ability to carry out functional activities.^26–29^ Yet despite their importance, research into how these “meaningful” symptoms and impacts map onto discrete DMOs has been underdeveloped. The Digital Measures that Matter to Patients framework is a widely recognised approach for evaluating the meaningfulness of individual digital measures.^21^ It begins by identifying a meaningful aspect of health based on the patients’ perspective; this is an aspect of a disease which the patient does not want to become worse, would like to improve or would like to present – typically a functional impact such as independence.^21^ This is then linked to a clearly defined concept of interest; this is a narrowed element of the meaningful aspect of health which can be practically measured, such as a symptom which impacts the meaningful aspect of health (e.g., shuffling gait). Through collaboration with domain experts and people with lived experience, an outcome that best captures this concept of interest is identified (e.g., stride length). Finally, an appropriate endpoint is specified, such as the degree of change in that outcome that is considered meaningful to patients.

Embedding the perspectives of people with lived experience is an important feature of this framework, ensuring their insights inform every stage of development; an element that is often lacking in digital health research in neurological disorders.^30^ It is important to include patients from diverse backgrounds and experiences to capture the needs and priorities of groups who have historically been under-served by research and healthcare, such as ethnic minority communities, people living in rural areas, and those with lower socioeconomic status. This is to ensure that existing health inequities are not further exacerbated.^31,32^ In a landscape where digital technologies make it possible to measure almost everything, incorporating diverse lived experience and establishing the meaningfulness of discrete DMOs ensures that outcome selection is guided by what matters to those living with the condition, not only by what is measurable.

The objectives of this study were to: (1) understand how motor and non-motor symptoms related to mobility (i.e., concepts of interest) impact meaningful aspects of health from the perspective of a diverse group of people with PD, inclusive of under-served groups, and (2) Identify “meaningful” DMOs by mapping the most impactful concepts of interest to discrete DMOs informed by the Mobilise-D core outcome set and current literature, guided by the Digital Measures that Matter to Patients framework.^21^ The findings are intended to inform one aspect of clinical outcome selection by providing evidence on patient meaningfulness, which should be considered alongside other important measurement properties, including technical and clinical validity and responsiveness to change.

## Methods

This international qualitative study included participants from the United Kingdom, Belgium, Germany and the United States. Ethical approval was granted by Newcastle University (ref: 2562/31981/2021), Central Bristol Research Ethics Committee (ref: 39/SW/025), the Ethics Committee Research UZ/KU Leuven, Belgium (Ref: S64977 and Ref: S67777) and the Ethics Committee within the Medical Facility of Kiel University (Ref: D475/23). Public and patient involvement (PPI) was embedded throughout every stage of the research process and is reported in line with current recommendations.^30^ A person living with PD (TGA) was a co-applicant on the grant to help prioritise the research focus; their activities are detailed throughout the methods and they were invited to comment on the final draft of this paper.

### Recruitment

The sample was derived using purposive sampling. Eligible participants self-reported a clinical diagnosis of PD, were aged ≥18 years and had capacity to provide voluntary informed consent. Participants were identified through a range of sources, including PD charities and voluntary organisations and charities, GDPR-compliant contact databases, existing research studies (i.e., the Mobilise-D cohort, East London Parkinson's Disease project), online sources including social media platforms, newsletters and forums, and via in-person engagement at PD support groups and exercise classes.

To maximise diversity and capture a wide range of experiences, we purposively recruited participants from a broad range of social, economic, environmental, and lifestyle contexts to maximise diversity within the study population.^31^ Our sample included people from ethnic minority backgrounds (predominantly South Asian), those with limiting conditions (e.g., co-morbidities such as poor vision and hearing loss), lower education and socio-economic status, those in later older age (80 + years) and those living rurally, coastally or otherwise remotely, guided by the CLEARS framework for digital exclusion.^33^ We also intentionally recruited groups that are historically under-represented in PD research and may have distinct experiences of mobility, including women, people with younger onset-PD, individuals in current employment, and participants identifying as LGBTQIA+.^34–37^

### Data collection

Participant demographics were collected prior to the interview, including age, gender, sexuality, co-morbidities, education and country of residence. Post codes or zip codes were used to estimate socioeconomic status (e.g., Index of Multiple Deprivation,^38^ Pobal HP Deprivation index,^39^ Area Deprivation Index,^40^ Northern Ireland Multiple Deprivation Measure^41^) and to classify rurality (e.g., Rural-Urban Classification,^42^ Am I Rural,^43^ Urban Rural Life^44^). Due to the qualitative nature of the study, clinical measures such as motor disease severity scores were not collected.

A semi-structured interview guide was developed, informed by literature examining meaningful aspects of health and related concepts of interest.^27,45–47^ The guide elicited participants’ experiences of living with PD, their self-described symptoms and the ways in which these symptoms influenced everyday life, including routine activities, employment, leisure, social participation and emotional wellbeing. For each symptom reported, participants were asked to elaborate on its impact, frequency and the strategies they used to manage it. They were then asked if they experienced common PD symptoms set out in a pre-defined list (see Appendix 1). Definitions of the symptoms were not provided by the researchers; participants drew on their own understanding of each symptom. Clarification was given only on request, which was generally limited to technical terms such as bradykinesia, and then conveyed in lay terms (e.g., “slowness of movement). Reported symptoms therefore reflect participants’ own recognition and experience rather than clinically assessed presence.

For all symptoms participants reported experiencing, they were asked to rate the extent to which each affected daily life on a scale from 0 (no disturbance) to 10 (extremely disturbing), used to calculate the bothersomeness score. The interview guide was piloted with a person with PD (TGA) using interpreter support and refined based on her feedback. This included identifying of questions that could be simplified, highlighting opportunities for additional prompts to facilitate more detailed questions, changes to the sequencing of questions to improve flow and including prefaces before symptom-related questions (e.g., “I’d like to focus on your symptoms and the effect they have on your daily life”). Additionally, TGA highlighted that interpreter support disrupted the flow of the conversation and made it difficult to maintain dialogue; as such, a small number of researchers were trained to conduct interviews in participants’ preferred languages across sites.

Interviews were conducted in the native tongue of the participant by a native speaking interviewer either in-person (where feasible), on an online communication platform, or on the telephone, according to participant preference. All participants provided informed consent. Average interview duration was 45 min (range: 19–109 min). All interviews were audio-recorded, transcribed verbatim and anonymised.

### Data analysis

Data collection and analysis took place in parallel, with emerging insights shaping subsequent interviews and informing ongoing refinement of the analysis. Data analysis involved two key stages: (1) identification of meaningful aspects of health and concepts of interest, and (2) mapping concepts of interest onto DMOs. This is illustrated in Figure 1 with definitions supplied in Appendix 2.

**Figure 1.**
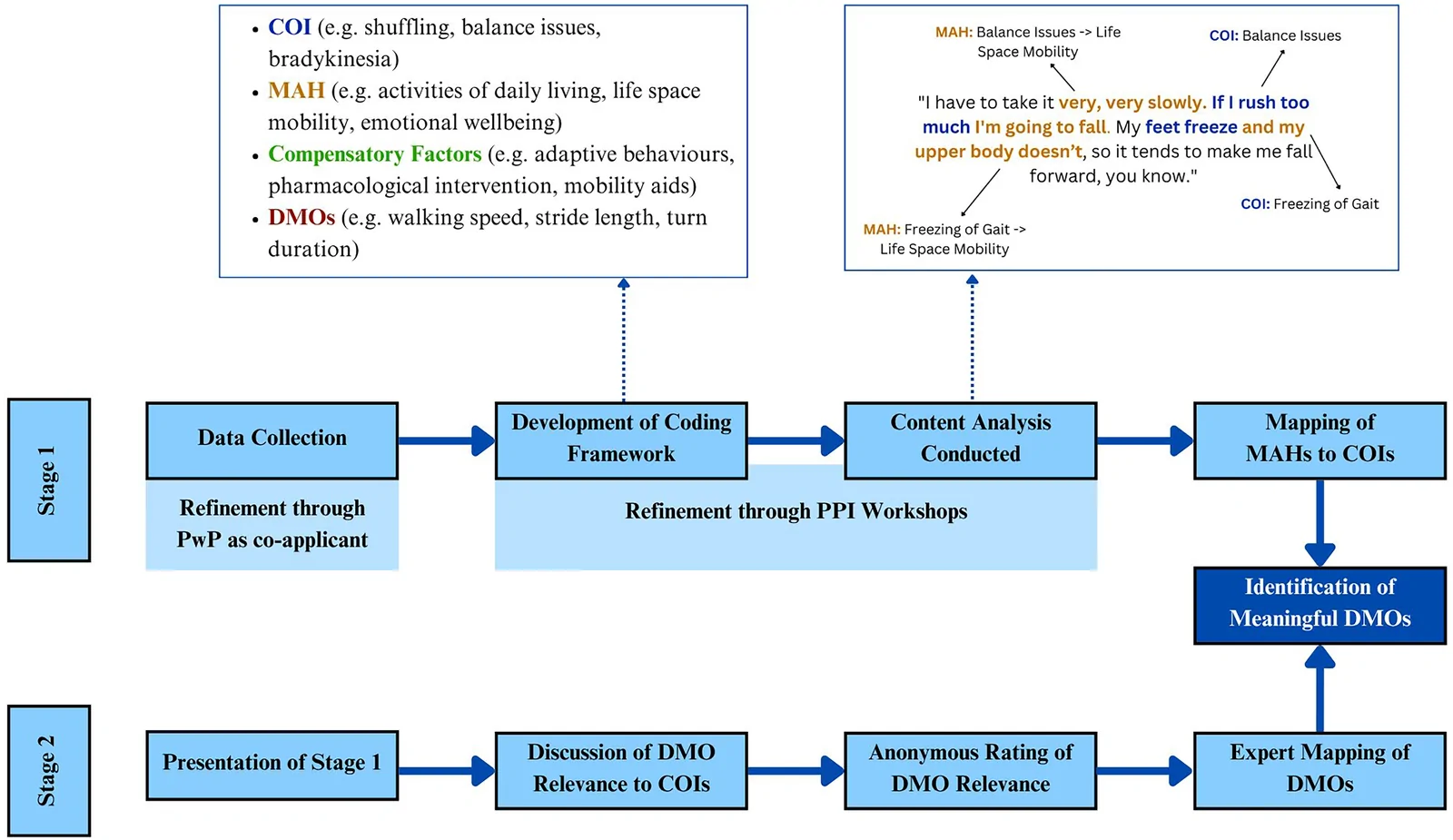
Protocol for the analysis of data. Abbreviations: MAH: Meaningful aspect of health; COI: Concept of Interest; DMO: Digital Mobility Outcome; PwP: Person with Parkinson's; PPI: Public Patient Involvement.

### Stage 1, identifying meaningful aspects of health and concepts of interest

Anonymised transcripts were imported into NVivo 14 and read and re-read for data familiarisation. A content analysis was used to focus on establishing patterns within the data.^48^ Following familiarisation of twenty transcripts, an initial coding framework was developed by JL, informed by the Digital Measures that Matter to Patients Framework,^21^ and focused on two primary constructs: meaningful aspects of health (coded as “impact”, e.g., emotional wellbeing) and concepts of interest (coded as “symptoms”, e.g., bradykinesia and rigidity).^21,45^ Associations between mobility limitations and both motor and non-motor symptoms were considered within analysis, and concepts of interest were categorised into symptoms which reported directly impacted mobility (hereafter referred to as mobility symptoms) and symptoms which did not directly impact mobility. The latter were not included in the analysis as this was beyond the scope of our aims (see Appendix 2 for details). Meaningful aspects of health were further categorised into activities of daily living, life space mobility and emotional impact (see Appendix 2 for definitions). Additional codes were devised to capture mediating or moderating factors between these two constructs (coded as “compensatory behaviours”, e.g., mobility aids). Instances of unclear or hard to interpret data were noted and then reviewed and discussed by the core research team to agree a consensus on coding (JL, MH, AK, RMA & KB). Numerical data were then exported into Microsoft Excel.

The proportion of the total sample experiencing each symptom was calculated and reported as frequency of symptoms. To establish how symptoms mapped onto meaningful aspects of health, the number of participants associating a symptom with an impact on activities of daily living, life space mobility or emotional wellbeing was identified. Proportional impact (%) was then calculated as:

$$\left(\frac{n\;reporting\;impact\;from\;symptom}{n\;experiencing\;symptom}\right)\times 100$$

This represents the conditional probability that a symptom affects a meaningful aspect of health among those who report experiencing it.

To align with previous research,^46,47,49^ the proportional bothersomeness score for each symptom was also calculated as follows:

$$\begin{array}{rl} & mean\;bothersomeness\;score\;of\;those\;experiencing\;symptom\;\\ & \;\times frequency\;of\;symptom(\text{\%}) \end{array}$$

Using Gephi software,^50^ these data were visualised into a network to better examine the relationships between the meaningful aspect of health (impact) and concept of interest (symptom), and the mediating or moderating factors. This involved exporting data into a matrix table on Microsoft Excel to determine the proportional impact onto each category for meaningful aspects of health.

Six PPI workshops involving 18 people with PD and 3 carers from under-served backgrounds led to data analysis plans being further refined. The coding framework (see Appendix 3) was refined to clearly and comprehensively reflect the experiences of people with PD, this included contributors expressing the importance of compensatory factors for mediating the walking experience which established this as a code going forward. Additionally, the map analysis was refined by discussing difficult to interpret quotes, which improved the clarity of analysis going forward. For example, contributors suggested going beyond literal quotes like “looking drunk” to infer this likely reflects issues with balance or dyskinesia.

### Stage 2, mapping concepts of interest to DMOs

A total of nine experts (Appendix 4) with complimentary technical and clinical expertise were recruited to participate in workshops aiming to map concepts of interest (symptoms) onto DMOs.^51–53^ These experts included neurologists, physiotherapists, biomedical engineers, psychologists, and sociologists, with combined expertise in PD, human movement science, digital health, digital mobility assessment and clinical trials. A nominal group technique approach was used.^53^ This involved a structured focus group in which experts discussed and identified how and why concepts of interest (i.e., mobility symptoms) should be mapped to each of the DMOs. The 24 DMOs that form the Mobilise-D technically valid core outcome set were chosen and categorised into broad categories to support interpretation (e.g., walking speed in walking bouts <30 s and walking speed in walking bouts > 60 s were categorised as “walking speed”).^13^ Following discussion amongst experts, it was highlighted that turning difficulties were frequently mentioned in participant transcripts and decided that “turn duration” should be included in the DMO set, as although this was not validated during Mobilise-D, it has been technically validated in PD and shows promise as a clinical marker within this condition.^15,54–60^ This left ten DMO categories (see Appendix 2). Experts then independently ranked the five DMOs they perceived as most relevant for each symptom (see Appendix 2). The aggregated independent rankings then fed into the map analysis by identifying which concepts of interest should map onto which DMO and the strength of each mapping, which was added to the visualised network to provide a conceptual understanding of the results.

## Results

### Participant demographics

Seventy-two people with PD participated in the study, with a median age of 64 years (range: 42–81 years). Most participants were white, living in the UK, and had been diagnosed with PD for an average of 5.8 years (range: 6 months to 20 years). Diversity was captured within the sample regarding age (3% in later older age), ethnicity (14% non-White), country of residence (including UK, Germany, Spain, Ireland, and USA), socioeconomic status (19% living in most deprived areas), rurality (31% residency), and LGBTQIA + (14% identified as gay). Further demographics are reported in Table 1.

**Table 1.** Participant demographics which highlight the number of participants in each under-served group.

Demographics	Participants (*N* = 72)
Gender (Women: Men), (*n*%: *n*%)	30: 42 (42%: 58%)
Age (median (min–max) years)	64.5 (42–81)
*≤50 years old, n* (%)	*9* (*13%)*
*≥80 years old, n* (%)	*2* (*3%)*
Length of Diagnosis (mean (SD, min-max) years)	*5.8 (3.9, 0.5–20)*
Ethnicity	
*White*	*62* (*86%)*
*South Asian*	*9* (*13%)*
*Arab*	*1* (*1%)*
Country of Residence	
*UK, n* (%)	*57* (*79%)*
*United States of America, n* (%)	*7* (*10%)*
*Germany, n* (%)	*6* (*8%)*
*Ireland, n* (%)	*1* (*1%)*
*Spain, n* (%)	*1* (*1%)*
Education	
*Compulsory Education (e.g., GCSE/O-Levels, equivalent)*	*6* (*9%)*
*Further education (e.g., A-Levels, BTEC, equivalent)*	*4* (*6%)*
*Higher education below degree (e.g., HNC, HND, diploma)*	*16* (*24%)*
*Undergraduate degree*	*15* (*23%)*
*Postgraduate degree*	*25* (*38%)*
Socioeconomic status area	
*Most deprived (1–3 IMD/equivalent)*	*14* (*19%)*
*Moderately deprived (4–6 IMD/equivalent)*	*27* (*38%)*
*Least deprived (7–10 IMD/equivalent)*	*31* (*43%)*
Rurality	22 (31%)
*Rural*	*22* (*31%)*
*Urban*	*50* (*69%)*
In employment, *n* (%)	12 (17%)
Sexuality	
*Straight, n* (%)	*62* (*86%)*
*Gay, n* (%)	*10* (*14%)*

Abbreviations: SD: Standard deviation; GCSE: General Certificates of Secondary Education; BTEC: Business and Technology Education Council; HNC: Higher National Certificate; HND: Higher National Diploma; IMD: Index of Multiple Deprivation.

### Identifying meaningful aspects of health and concepts of interest

Most participants (*n* = 70/72; 97%) reported experiencing motor and non-motor symptoms associated with reduced mobility, with the most frequently reported symptoms being shuffling (86% of participants), bradykinesia (76%) and balance issues (74%). The most proportionally bothersome symptoms reported were shuffling (proportional bothersomeness: 5/10), bradykinesia (4/10) and balance issues (4/10), reflecting frequency of symptoms. Frequency and bothersomeness ratings can be found in Figure 2 (for scores of overall group and stratified by time since diagnosis, see Appendix 5 and 7).

**Figure 2.**
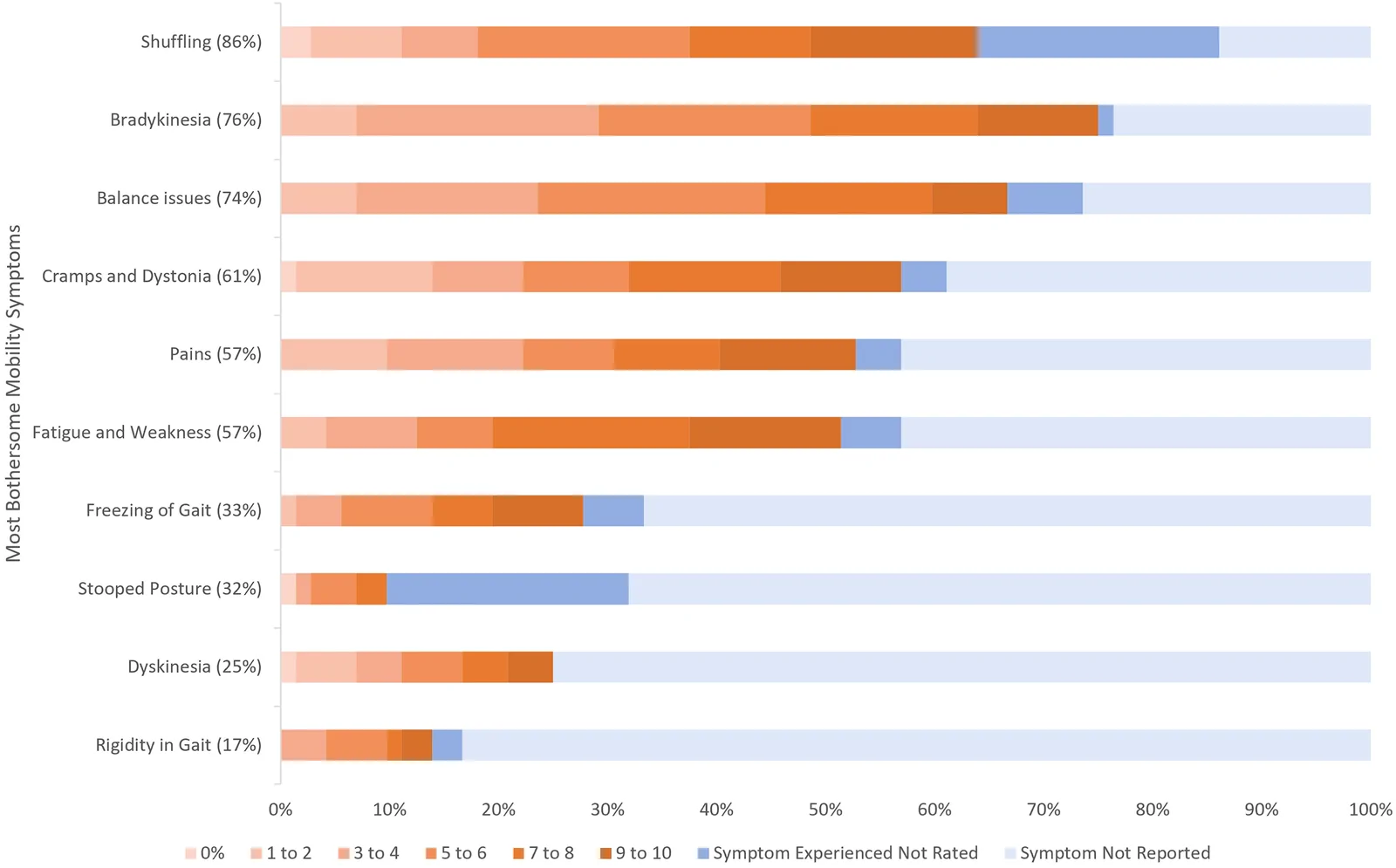
Frequency and bothersomeness of mobility symptoms experienced by participants.

Of the 72 participants, 97% associated at least one physical mobility symptom with at least one meaningful aspect of health, with 86% reporting impacts on impairments in activities of daily living, 71% on reduced life space mobility and 56% on worsening emotional wellbeing. These associations will be discussed below for the most frequently reported, impactful, bothersome mobility symptoms. Supporting quotes from participants will be provided with markers of age, country of residence, time since diagnosis and any under-served characteristics that they represent.

#### Shuffling

Shuffling was the most frequently reported and proportionally bothersome symptom. Of the 62 participants who reported shuffling while walking, 52% associated this with impacts on meaningful aspects of life. 32% felt that shuffling gait impaired their ability to carry out activities of daily living by increasing the time and energy required to move around the environment.

*“I’ll shuffle out of bed and shuffle to take my meds, shuffle to let the dogs out, then I’ll shuffle to the living room and just sit until my meds come on, because it's not safe for me to do anything like get ready for work. It's not safe for me to take a shower until I’m on.” P14 (45-year-old female, deprived area in the USA working full-time, 3 years since diagnosis)*

24% associated shuffling with reduced life space mobility, particularly as it heightens risk of falls. 21% linked reduced emotional wellbeing to shuffling, as they experienced both internalised and externalised stigma due to the visible nature of the symptom.

*“I was dragging the leg at this stage. So, I looked like a right fecking monster of some sort of (Laughter) Frankenstein.” P40 (49-year-old male, Ireland working full-time, 5 years since diagnosis)*

35% of these participants demonstrated compensatory behaviours, such as exercising caution and consciousness when stepping.

*“Well, I hear it, and it doesn’t make me very happy because it means that the disease is progressing, and I try to pick up my feet and definitely keep my head up because when you hear that and you’re going slow, you tend to look down so that you don’t trip on anything.” P51 (64-year-old female, rural USA, 12 years since diagnosis)*

#### Bradykinesia

Bradykinesia was the second most frequently reported mobility symptom, reported by 55 participants. 64% of participants experiencing bradykinesia attributed the symptom to impacts on meaningful aspects of health. Within this, 53% described bradykinesia leading to impairments in activities of daily living, mostly commonly because the symptom restricted what an individual could accomplish in a day.

*“I can cook dinner, if I really have to, but it takes me about an hour-and-a-half to do what [my husband] could do in 20 min.” P65 (57-year-old female, rural UK, 3 years since diagnosis)*

24% associated bradykinesia with diminished emotional wellbeing, as they felt that the symptom reduced their ability to stay independent and left them increasingly socially isolated.

“*My world has become smaller and is in danger of becoming even smaller because I can’t get out and meet people and socialise properly.” P93 (61-year-old LGBTQIA* *+* *male, deprived area in the UK, 3 years since diagnosis)*

The physical manifestations of bradykinesia in which individuals became visibly slow within their movements also led to experiences of externalised stigma, due to misconstrued perceptions from others.

“*Probably the slowing down, just because it's the more visible thing. People are going to notice that you… My wife said to me the other day, “You look like you’ve had one too many today.”” P1 (70-year-old male, rural UK, 11 years since diagnosis)*

While participants associated bradykinesia with reduced abilities to carry out activities of daily living and worsened emotional wellbeing, the symptom did not appear to impact life space mobility with only one participant reporting a link between the two. 29% of participants compensated for their bradykinesia by alternating the timing of their medication regimens or activities so that they could avoid this symptom when they needed to perform certain activities.

“*When the medication is working well, I can do most things that I used to be able to do. So, preparing food, doing manual tasks, working around the house, that kind of stuff. When the medication is worn off, it's slow.” P18 (61-year-old male, UK, 6 years since diagnosis)*

### Balance issues

Balance issues were the third most frequently reported mobility symptom (*n* = 53), of which 45 participants described these as impacting a meaningful aspect of health, constituting the largest proportional impact among the symptoms examined. Within this, 76% associated balance issues with reduced life space mobility, encountering experiences of falls and difficulties navigating stairs.

“*I haven’t fallen, but my balance isn’t as good as it used to be, that's impaired. Stepping around things, objects, accidentally brushing up against the wall, do you know what I mean, or somebody near you? Uneven ground, I tend to have to pick my way through that, so I go from slow to ultra slow, that's balance.” P69 (73-year-old male, UK, 2.5 years since diagnosis)*

57% experienced impairments in carrying out activities of daily living, including completing household tasks such as cooking and cleaning, carrying out personal care activities such as bathing and dressing, and participating in leisure activities like gardening and hiking. 25% associated balance issues with worsening emotional wellbeing, reporting erosion of confidence, increased reliance on others, and feelings of embarrassment in oneself.

“*If I'm going to take a shower, I always need to make sure I'm holding on. The problem with Parkinson's is having to rely on a walking stick to avoid falling. My balance isn’t what it used to be” P117 (75-year-old-man, rural Belgium, 20 years since diagnosis)*

“*[The balance issue] slows me down, and it makes me uncertain of myself. One of the big issues I find with having Parkinson's is confidence, it can destroy your confidence if you’re not careful” P2 (60-year-old male, UK, 2 years since diagnosis)*

43% reported using mobility aids, while 30% adapted their behaviours to support their balance, such as cautiously slowing down while walking and becoming more conscious of their movement.

“*Well, it's just when you’re out, if you want to turn or anything, you’ve got to be really careful. I'm very careful so I don’t fall. I have to hold on to my husband because I could go off balance very easily.” P61 (81-year-old female, UK, 7 years since diagnosis)*

#### Less frequently reported mobility symptoms

While other mobility symptoms were less frequently reported by participants, some were still bothersome and had a notable impact on meaningful aspects of health for those who experienced them. Within this section, we will highlight three less prevalent symptoms with high proportional impacts. Appendix 6 and 7 provides percentages of proportional impact for each mobility symptom for the overall group and by time since diagnosis.

56% (*n* = 41) of participants experienced fatigue and weakness, considered a physical mobility symptom due to its impact on physical capacity. Of those participants, 56% reported these symptoms impacting meaningful aspects of health; primarily by impairing abilities to carry out activities of daily living (experienced by 46%) with limited impact on life space mobility and emotional wellbeing (impact experienced by 5% and 12% respectively).

“*I feel more tired and feels like I have no energy. For example, after I wake up, go to the toilet and shower and get ready, after that I have no energy left in me. Like yesterday I only made lentils all day because I had no energy in me left.” P104 (59-year-old South Asian female, UK)*

Experiences of freezing of gait were reported by 33% of participants (*n* = 24), with 42% reporting an impact on meaningful aspects of health. This was primarily emotional wellbeing (25%), while 17% reported impacts on life space mobility and emotional wellbeing.

“*The freezing because it means I can’t mobile as I like to be. I used to be very active and now I am not active anymore. I find that really pisses me off. Sorry, but it does.” P35 (73-year-old male with limited formal education, UK, 5 years since diagnosis)*

Only 25% (*n* = 18) of participants reported experiencing dyskinesia. However, for these, dyskinesia demonstrated a high proportional impact with 50% associating it with an impact on a meaningful aspect of health. Within this, 28% experienced challenges carrying out activities of daily living, and reduced emotional wellbeing due to experiences of external stigma.

“*I just don’t feel safe and steady enough and comfortable enough, and obviously, if I’ve got the dyskinesia going on or I’m not walking too well or whatever, then I feel like everybody is looking at me and wondering what the hell is going on or judging me or thinking I’m drunk, on drugs or it's not normal.” P50 (45-year-old LGBTQIA* *+* *male, UK, 5.5 years since diagnosis)*

### Mapping concepts of interest onto DMOs

The mapping analysis of the most impactful, bothersome, and prevalent mobility symptoms has been visualised in Figure 3A. Figure 3B highlights walking speed, turn duration, and stride length as the most relevant DMOs associated with the most impactful and bothersome concepts of interest as identified during expert workshops. Table 2 reports the top five DMOs relevant to each mobility symptom according to expert consensus. The combined individual rankings can be seen in Appendix 8.

**Figure 3.**
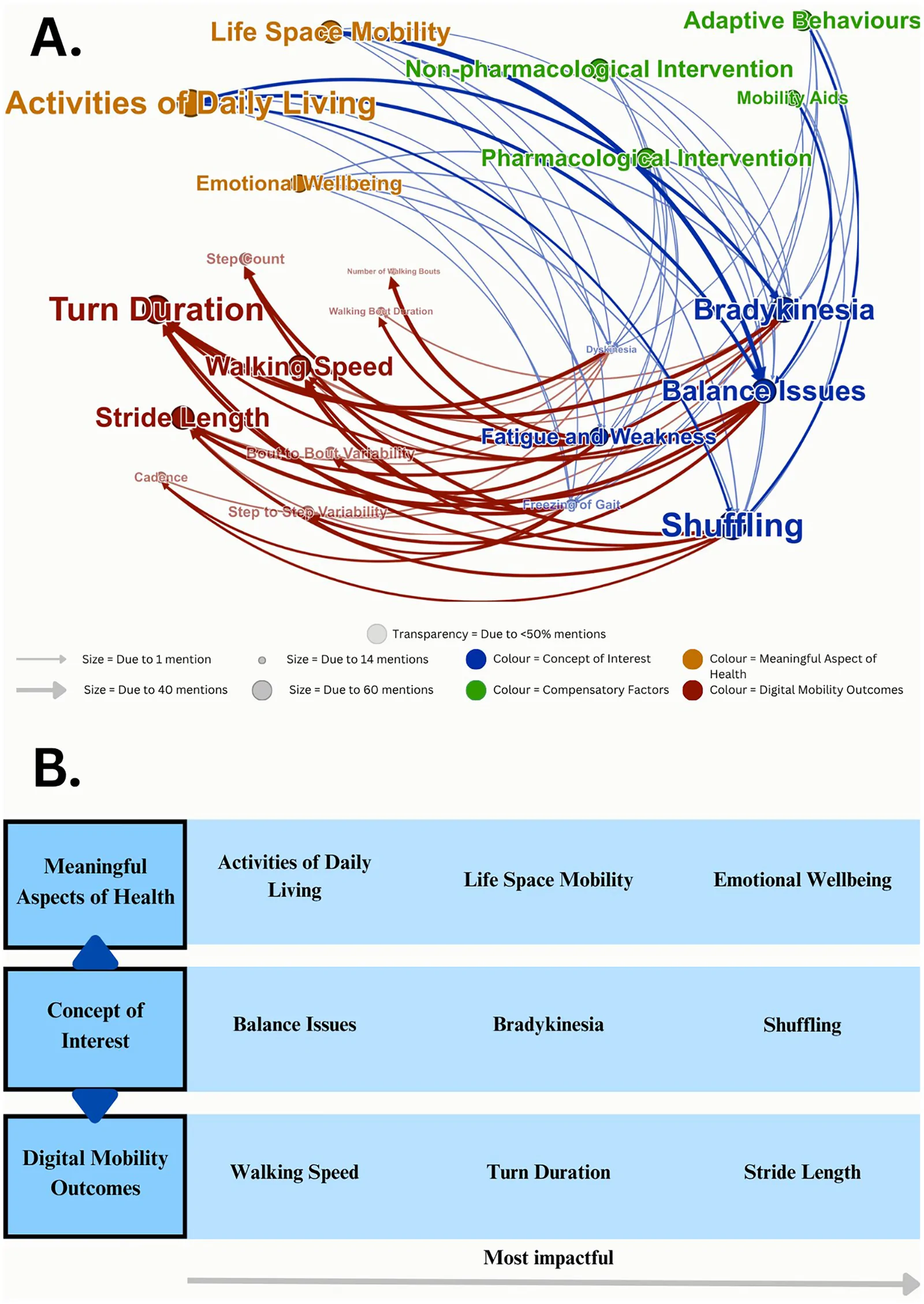
Conceptual map of participant experiences and meaningfulness of digital mobility outcomes. Figure 3A depicts a conceptual map of participant experiences, visualising how meaningful aspects of health and compensatory factors affect concepts of interest, and how this can be measured using digital mobility outcomes. Figure 3B highlights the most impactful meaningful aspects of health and concepts of interest, and the digital mobility outcomes which best measure these.

**Table 2.** Consensus of the mapping from symptom to DMO.

Symptoms	Digital mobility outcomes
Balance Issues	1. Stride Length
	2. Step to Step Variability
	3. Walking Speed
	4. Turn Duration
	5. Bout to Bout Variability
Bradykinesia	1. Turn Duration
	2. Stride Length
	3. Cadence
	4. Walking Bout Duration
	5. Walking Speed
Fatigue and Weakness	1. Step Count
	2. Number of Walking Bouts
	3. Bout to Bout Variability
	4. Walking Bout Duration
	5. Walking Speed
Shuffling	1. Step Count
	2. Turn Duration
	3. Stride Length
	4. Walking Speed
	5. Cadence
Dyskinesia	1. Turn Duration
	2. Walking Speed
	3. Stride Length
	4. Step to Step Variability
	5. Bout to Bout Variability
Freezing of Gait	1. Walking Speed
	2. Turn Duration
	3. Cadence
	4. Step to Step Variability
	5. Stride Length
Cramps and Dystonia	1. Walking Speed
	2. Number of Walking Bouts
	3. Walking Bout Duration
	4. Step Count
	5. Volume of Activity
Pains	1. Walking Speed
	2. Number of Walking Bouts
	3. Walking Bout Duration
	4. Step Count
	5. Volume of Activity
Rigidity in Gait	1. Turn Duration
	2. Walking Speed
	3. Stride Length
	4. Cadence
Movement Imitation	1. Stride Length
	2. Walking Speed
	3. Turn Duration
	4. Cadence
	5. Step to Step Variability
Stooped Posture	1. Cadence
	2. Stride Length
Arm Swing	1. Walking Speed
	2. Step to Step Variability
Asymmetry of Motor Symptoms	1. Turn Duration
	2. Walking Speed
	3. Step to Step Variability
	4. Stride Length
	5. Cadence

Note: Symptoms are in order of proportional impact and DMOs are in order of relevance.

## Discussion

The aim of this study was to identify meaningful DMOs by understanding how PD symptoms related to mobility impact aspects of health that matter to people with PD, directly addressing regulatory guidance emphasising the need for lived experience to shape the selection of meaningful outcomes implemented in clinical trials.^12,21^ A novel aspect is the intentional inclusion of under-served groups, including those at heightened risk of digital exclusion,^33^ whose experiences are rarely represented in PD research.^61^ Key findings suggest that balance issues, shuffling, and bradykinesia were the most frequently reported and meaningful symptoms related to mobility across the sample, while experiences of freezing of gait, fatigue and weakness were less frequently reported but considered to be highly impactful in those who did experience them. Real-world DMOs were mapped to all symptoms by an expert group to provide a framework for the digital and patient-relevant assessment of mobility in PD. The three most meaningful DMOs were determined as walking speed, stride length and turn duration as they mapped to the most meaningful symptoms reported, as well as less frequently but still highly impactful symptoms such as freezing of gait and dyskinesia.

Our key findings suggest that balance issues, shuffling, and bradykinesia were the most frequent, bothersome and impactful mobility symptoms experienced in PD (proportionate to those who reported them), contributing to reduced abilities to carry out activities of daily living, restricted life space mobility and diminished emotional wellbeing. Previous research has consistently reported motor symptoms to be the most pervasively bothersome across all disease stages in PD, particularly those related to mobility limitations such as gait and balance impairments.^26–28,49,62^ Mobility limitations are often cited as increasingly important as the disease progresses with significant impacts on physical and psychosocial functioning.^27,28,46^ To some degree, this is reflected in our data whereby descriptive data highlights bradykinesia and balance issues as most bothersome and impactful to those diagnosed with PD for more than 10 years; however a dedicated longitudinal study with a large sample would be required to draw conclusions about this. Recently, movement disorders specialists postulated that a focus on specific symptoms rather than the broad concept of mobility limitations would have greater potential for use in the selection of outcomes for clinical trials in PD,^63^ while a systematic review has highlighted that there is little available data investigating the meaningfulness of specific mobility symptoms.^26^ Our work addresses this gap by identifying specific symptoms related to mobility and linking these to impacts on meaningful aspects of health. This enables triangulation across discrete symptoms, lived experience, and functional outcomes. However, we note that it was conceptually difficult for participants to disentangle discrete symptoms from their overall experience of mobility limitations in PD, as multiple symptoms contribute simultaneously to meaningful aspects of health. For some, it was this broader sense of declining mobility that was most distressing. While our analysis enabled interpretation of these experiences in relation to common symptom profiles, the use of bothersomeness scores relied on participants being able to identify and reflect on discrete symptoms based on their understanding of them.^43,44,46^ As this understanding may not always align with clinical definitions, reported symptoms reflect lived rather than clinically assessed experience and are sensitive to how each symptom is interpreted; bradykinesia, for instance, is an essential criterion for the diagnosis of PD,^3^ yet some participants did not report experiencing it at all. However, the capacity to recognise and interpret symptoms may be more difficult for some, particularly where insight shaped by wider systemic and societal barriers.^64^ More inclusive approaches may be needed to capture what is most meaningful for individuals whose opportunities to understand and interpret their symptoms are shaped by inequalities in access to information, clinical support, and time with healthcare professionals.

The top three meaningful DMOs identified were walking speed, stride length and turn duration, based on an expert consensus of relevance to the most bothersome and impactful mobility symptoms. While multiple studies have now identified meaningful symptoms for people with PD,^26–28,49,62^ few have mapped these concepts of interest onto digital measures. Where this has been done, people with PD have usually been asked if they feel that an instrumented task capturing broad digital measures, such as “walking and balance”, is relevant to their symptoms and impacts, as opposed to the discrete DMOs employed within this study.^46,47^ For example, during the baseline assessment of the WATCH-PD study, eight clinic-based tasks captured were endorsed as personally relevant by the participants with early-stage PD, including various movement, speech and cognitive measures.^47^ After three years, these broad categories of tasks all remained personally relevant, although there was an observed increase in the reported relevance of the walking and balance task (captured by a smartwatch).^46^ Additionally most participants suggested these tasks should be more challenging to reflect the lived experience of PD. Our findings extend this work by identifying discrete DMOs which can be captured in real-world settings, allowing greater ecological validity of the challenges people with PD may face in daily life.^65^

These three DMOs have additionally been associated with relevant clinical aspects of PD. Walking speed is the most common real-world DMO captured in PD, and people with PD consistently demonstrate slower walking speeds and shorter stride lengths than age-matched controls.^66^ Walking speed and stride length are also reduced in later stages of PD compared to earlier stages, suggesting they reflect disease progression.^24^ Real-world turn duration is a relatively more novel DMO representing an essential and relevant component of mobility as ∼45% of real-world gait is composed of turning.^67^ Turning is a complex movement, requiring a high degree of inter-limb co-ordination and step asymmetry, and cognitive input; as such, it is often the trigger for freezing of gait and falls in PD.^59^ Slower turn duration has been associated with complex cognitive functions and increased fall risk in people with PD.^57,58^ Early evidence indicates that these three real-world DMOs are sensitive to the effect of current PD medications, with reports of faster walking speeds, longer strides and reduced turn duration when “ON” medication, supporting their application as outcomes for clinical trials.^68,69^ We envisage the results of this study to support the selection of digital measures for future clinical trials, as our approach focuses on DMOs that have already undergone technical validation with emerging evidence of clinical validity.^9,13,24^ However, patient meaningfulness only reflects one aspect relevant to decision making regarding outcome selection. Importantly, selection should also be guided by context of use, reflecting what researchers and clinicians need to interpret and act on in practice, and the ability of outcomes to detect clinically meaningful change over time in response to disease progression or treatment. For this reason, we presented the top five DMOs for each mobility symptom, recognising that outcome selection should be tailored to the specific clinical trial context and informed by both patient meaningfulness and measurement performance.

Our approach is based on the recognised Digital Measures that Matter to Patients framework to evaluate the meaningfulness of individual digital measures.^21^ Additionally, although we took a less individualised approach, our quantification of qualitative data broadly emulates the work of Mammen,^47^ in which they employed a symptom-response card sorting technique to map associations between symptoms and meaningful aspects of health. These quantified approaches offer a useful, tangible structure for selecting digital measures in both research and clinical settings. However, they do not fully capture the dynamic and individualised nature of how symptoms are experienced and managed in everyday life.^47,70^ The relationship between single concepts of interest and meaningful aspects of health is not linear. For example, one of our participants described how her stooped posture affected her walking, caused pain and made her feel self-conscious, resulting in less time spent outside the home. A stooped posture is often associated with additional complications around stiffness, pains, and difficulty walking, which then discourages other activities. This highlights the complex interplay between mobility symptoms and compensatory behaviours which interact to shape multiple, interdependent health outcomes.^46^ Furthermore, constructing associations between symptoms and meaningful aspects of health may not be a “one-size-fits-all” approach. In our sample, participants from under-served groups faced additional barriers which impacted meaningful aspects of health. For instance, participants living in rural areas described how mobility symptoms intersect with more challenging environments, such as uneven paths, heightening concerns about falling and further restricting their life space mobility. Likewise, quantitative measures of the frequency, bothersomeness or proportional impact of symptoms on meaningful aspects of health do not illustrate how dynamically participants experience these symptoms and impacts. These associations can change according to the disease stage or acceptance over time, and they can be dependent on medication timing.^26,27,46,47,49^ To ensure we are capturing the full breadth of lived experience when selecting meaningful measures, we should also consider the contribution that rich qualitative data can have in identifying the meaningfulness of broad domains of mobility assessment, such as the quantity versus quality of mobility.

### Strengths and limitations

A key strength of this study is the purposeful recruitment of under-served groups, given the marked lack of diversity across PD research internationally, allowing for a large and representative dataset.^61^ Rather than looking at distinct experiences of discrete under-served groups (e.g., ethnic minorities), our priority was to ensure these typically excluded from research directly shaped a shared account which can apply broadly to clinical trials in PD, reflecting our initial aims and intent on inclusive recruitment. While capturing these distinct experiences is important to meet the needs and priorities of specific groups, this requires a dedicated study with larger sample sizes of each group; we recommend future research addresses this area in line with the call to action to improve diversity and inclusivity in PD research and care.^61^ This study was strengthened by the PPI contributions throughout the research cycle, including the priority setting, study design and interpretation of data analysis. This ensured the methods used and conclusions drawn are relevant to the experiences of people with PD and supported the use of an exploratory mapping approach between concepts of interest, meaningful aspects of health and DMOs, by triangulating multiple experiences to develop consensus.^30,51–53^

This study is not without limitations. While our community-based approach to recruitment allowed us to capture a more diverse range of viewpoints, it also prevented us from acquiring formal clinical information regarding diagnosis or disease staging and severity. Our reliance on self-reported data therefore did not allow us to compare people with milder stages of PD compared to moderate and severe, which may have affected the prevalence rates of the symptoms and changed our interpretation of the meaningfulness results.^27,49^ We only included people with PD in this study, whereas the inclusion of carers may have provided different perspectives as the impact of mobility challenges in PD are often experienced dyadically.^70^ Aligned with our aim to identify meaningful DMOs, we only included PD symptoms which directly related to mobility in our analysis. Yet, we acknowledge that presence of a resting tremor was a frequently experienced and bothersome symptom for our participants, and while this may not be captured by DMOs, other digital measures may be useful to capture meaningful non-mobility symptoms.^46,47^ Additionally, we included turn duration in our mapping approach despite not being part of the Mobilise-D core outcome set. This decision was due to our experts’ perception of the clinical importance of this emerging DMO and external technical validation efforts; however, we note this as a limitation as it has not received the same scrutiny as those within the Mobilise-D set.^13,54^ Finally, although we purposively sampled for diversity, all participants lived in high-income Western settings with the majority based in the UK. Meaningfulness of mobility symptoms may be shaped by wider cultural, societal and systemic influences, and these results may not be globally representative. For example, one study reported that mobility impairments meant people with PD in East Africa could not uphold societal expectations, such as standing to greet a visitor, which may contribute to social isolation and diminished wellbeing.^71^ In the development of an Activities of Daily Living assessment for older adults in rural India, mobility was considered less relevant than self-care, social participation, and cognitive functioning, reflecting the roles and responsibilities typically undertaken by older adults within that sociocultural context.^72^ Given this nuance, we recommend further research is undertaken to understand the meaningfulness of mobility in PD beyond the Western context, and to consider how this influences the selection of DMOs in different settings.

## Conclusion

This study is the first to offer a framework to support the selection of meaningful DMOs for clinical trials in PD, inclusive of under-served groups and developed with PPI across the research cycle. Further work should consider more equitable approaches to developing conceptual frameworks for meaningful digital mobility measures, ensuring that the voices of those with reduced access to information about their conditions are fully included.

## Supplemental Material

sj-docx-1-pkn-10.1177_1877718X261494917 - Supplemental material for Meaningful digital mobility outcomes for people with Parkinson's disease: Insights grounded in diverse lived experience Supplemental material, sj-docx-1-pkn-10.1177_1877718X261494917 for Meaningful digital mobility outcomes for people with Parkinson's disease: Insights grounded in diverse lived experience by Jack Lumsdon, Ríona Mc Ardle, Megan Hanrahan, Alison Keogh, Cameron Wilson, Tova Gur Arieh, Alastair J Noyce, Essa Bhadra, Johanna Graeber, Lova Hulst, Moran Gilat, Heiko Gassner, Jürgen Winkler, Walter Maetzler, Anat Mirelman, Alison J Yarnall, Lynn Rochester and Katie Brittain in Journal of Parkinson's Disease

## Data Availability

The data collected and analysed within this study are available upon request from the corresponding author.
