# Supplementary material for: Meaningful digital mobility outcomes for people with Parkinson's disease: Insights grounded in diverse lived experience
Source: J Parkinsons Dis. 2026 Oct 9:1877718X261494917. Online ahead of print. doi: 10.1177/1877718X261494917 (PMC13652976; doi:10.1177/1877718X261494917)
Supplement: Supplementary file 1 [file sj-docx-1-pkn-10.1177_1877718X261494917.docx]

## Supplementary materials

### **Appendix 1.** Pre-defined symptom list to prompt participants during the interview

| **Symptoms** | **Yes/No** |
| --- | --- |
| Tremors and dexterity, fine motor movements |  |
| Slow movement (bradykinesia) |  |
| Muscle stiffness (rigidity) |  |
| Muscle cramps |  |
| Balance issues |  |
| Fatigue, tiredness, weakness |  |
| Freezing movements (freezing of gait) |  |
| Pain in your neck, chest, back, arms, legs or feet |  |
| Shuffling walk, dragging of the foot |  |
| Involuntary movements (dyskinesia) |  |

*Note: This list was developed alongside a person with Parkinson’s to be easily understood by participants and to prompt participants to discuss symptoms during the interview.*

### **Appendix 2.** Glossary of data analysis

| **Experience** | **Definition** |
| --- | --- |
| **Meaningful aspect of health** | **An aspect of disease that a patient does not want to become worse or wants to improve or wants to prevent (1)** |
| **Activities of Daily Living** | **Activities orientated towards taking care of one’s own body; these are fundamental to survival. Instrumental Activities of Daily Living are also included under this code, which focus on activities which support daily life within the home and community, often requiring more complex interactions (2)** |
| Requiring Assistance | Needing the help of family/friends/carers to carry out tasks. Including if they need to adapt their behaviours to support and enable the person with Parkinson’s. |
| Household Activities | Impacted ability to do chores relating to maintaining a household such as cleaning, cooking, DIY, and shopping |
| Personal Care Activities | Impacted ability to do tasks relating to personal care such as bathing, dressing, and eating |
| Leisure Activities | Impacted ability to carry out leisure activities such as hobbies, gardening, and sports (including walking for leisure) |
| Social Activities | Impacted ability to carry out social activities such as meeting friends and going to support groups |
| Occupational Activities | Impacted ability to carry out activities relating to their work (both employment and voluntary) whether this is keyboard use, retirement |
| Travel and Transport | Impacted ability to travel, both in terms of locally and internationally |
| **Life Space Mobility** | **How an individual moves across the physical and social environments they inhabit on a day-to-day basis (3)** |
| Difficulty Turning | Slow and unsteady turns |
| Increased Danger | Increased risk of danger in the absence of a fall, such as bumping into things, dropping things, difficulty crossing a road safely |
| Needing to Plan | Planning the day around the symptoms, such as timing certain activities around on and off states |
| Cautiously Slowing Down | Slowing down the pace of activities due to feeling cautious and reduced confidence |
| Difficulty with Certain Environments | The environment influencing the individual’s mobility such as difficulty with stairs, uneven terrain, etc |
| Difficulty Transferring | Impaired ability to transfer, including in and out of the car, a bath, or sit to stand (in situations whereby Movement Initiation does not apply) |
| Falls | An unintentional or unexpected event that results in the person being on the ground or another low level, and is not the result of an intrinsic event or external force (4) |
| **Emotional Wellbeing** | **How an individual feels generally about life overall, including both experiences and reflections (5)** |
| Emotional Impact | Impacted general wellbeing such as feeling embarrassed, decreased confidence, and feeling emotional |
| Mental Health Struggles | Impacted mental health such as feelings of anxiety and depression |
| Shrinking World | Feeling of the world closing in, hesitant to explore and do new and difficult things |
| Stigma | Referring to both self and external stigma experienced as well as feelings of embarrassment |
| Fear of Falling | Strong feelings of fear towards falling |
| **Concept of Interest** | **A measurable element of a meaningful aspect of health, such as a symptom which most impacts that aspect of health (1)** |
| **Mobility Symptoms** | **Symptoms directly affecting how an individual is able to freely move around the environment (6)** |
| Balance Issues | Loss of control of the body’s centre of mass over its base of support to achieve postural equilibrium and orientation (7). Participants experience this as losing stability, feeling as though they may fall |
| Stooped Posture | Abnormally flexed body posture with a forward bending posture from the middle or lower back, forward lean of the head and neck, and a lateral curve of the spine (8) |
| Cramps and Dystonia | Muscle cramp is the tightening of the muscle (9) and dystonia is a sustained muscular contraction frequently accompanied by abnormal movements and postures (8). Participants often associate these symptoms with pain |
| Pains | Painful sensations affecting musculoskeletal and neuropathic domains, this can involve primary and central parkinsonian pain (9). This was described by participants as pains, aches, and soreness. |
| Freezing of Gait | Brief episodes of inability to step or by extremely short steps that typically occur on initiating gait or while turning (10) |
| Arm Swing | Arm swing reduction and asymmetry while walking (11) |
| Dyskinesia | Involuntary movements which occur at peak medication concentrations, such as bodily swaying (12) |
| Movement Initiation | Difficulty moving after a period of inactivity, often taking participants longer to begin movement (13) |
| Rigidity in Gait | Involuntary resistance to passive movement (12), this manifests as muscular rigidity and stiffness impacting upon walking |
| Bradykinesia | Reduced velocity of movements (14). Participants experienced this as slowness in walking and everyday activities. |
| Shuffling | Scuffing/dragging of the foot along the floor while walking, either a slow short-stepped gait pattern or festination (rapid, small steps, done in attempt to keep balance) (15, 16). |
| Asymmetry of Motor Symptoms | Symptoms affecting one side of the body more severely than the other |
| Fatigue and Weakness | Feeling tired and unable to continue activities, as well as being too weak to complete an activity |
| **Non-Mobility Symptoms** | **Symptoms which do not directly affect an individual’s ability to freely move around the environment, although these symptoms may indirectly affect mobility.** |
| Pins and Needles | A temporary tingling, prickling, or numbness sensation |
| General Rigidity | Muscular rigidity and stiffness not relating to mobility, such as a stiff neck, facial masking, and difficulty turning in bed |
| Autonomic Symptoms | Symptoms which relate to cardiovascular, gastrointestinal, thermoregulatory, pupillomotor, and sexual functioning (17). This includes symptoms relating to bodily fluids and functions such as drooling, sweating, blood pressure, change in smell, and bowel problems. Urinary Urgency was separated due to the it’s distinct impact and importance to participants. |
| Urinary Urgency | Increased urgency and frequency of urination (17) |
| Tremors and Dexterity | Typically a resting pill-rolling type of tremor of the hands and difficulty with fine motor movements, as well as difficulty writing (8) |
| Voice Related Symptoms | Symptoms relating to speech such as low or slurred speech |
| Sleep Difficulties | Symptoms of rapid eye movement sleep behaviour disorder, daytime sleepiness, and sleep maintenance insomnia (12). Experienced by participants as difficulty falling and staying asleep, such as insomnia |
| Cognitive Impairment | Difficulty with cognition such as executive function (planning, working memory, shifting attention) (18) |
| Restless Legs | Urge to move one’s legs, often accompanied by an uncomfortable or unpleasant sensation in the legs, which is usually worse during periods of inactivity (19) |
| **Compensatory Factors** | **Factors mediating and managing symptoms to reduce the impact on daily living** |
| Pharmacological Intervention | Primarily dopaminergic medication which is used to manage symptoms, and typically results in ‘on’ and ‘off’ states as the condition progresses (12) |
| Non-Pharmacological Intervention | Non-pharmaceutical interventions such as psychological/physical therapy, exercise, Botox injections, and deep brain stimulation |
| Adaptive Behaviours | Adapting behaviours to modify symptoms such as compensatory behaviours (changing the way they do the activity, and grabbing onto the walls for support), persevering through activities, consciously modifying their gait, etc |
| Mobility Aids | Use of mobility aids such as walking sticks, and trolleys |
| **Digital Mobility Outcomes (definitions adapted from Lumsdon et al (20) and Mazzà et al (21))** | **A measure of mobility quantified from a wearable device which can measure a Meaningful Aspect of Health through a relevant Concept of Interest (1, 22)** |
| Walking Speed | Distance covered by the whole body within a certain time interval per unit of time walking. It is measured in meters per second and is the magnitude of the velocity vector. Defined as the daily average measured across all walking bouts. |
| Stride Length | The interval between two successive initial contacts of the same foot. Thereby, every stride contains two steps. Defined as the daily average measured across each walking bout. |
| Cadence | The number of steps taken per minute. Defined as the daily average measured across each walking bout. |
| Turn Duration | The time between the start and the end of the turns within the walking bout. |
| Step to Step Variability | Difference in the pace and rhythm between steps within a walking bout. |
| Walking Bout Duration | Duration between the start and the end of a walking bout. Defined as the daily average measured across each walking bout. A walking bout is a walking sequence containing at least two consecutive strides of both feet. The start and end of a walking bout are determined by a resting period or any activity (non-walking period). |
| Number of Walking Bouts | Number of walking bouts taken per day. |
| Bout to Bout Variability | Difference in the pace and rhythm between walking bouts. |
| Step Count | Number of steps taken per day. |
| Amount of Activity | Amount of time spent walking. |

### **Appendix 3.** Demographics of PPI contributors

| Demographics | Participants (N = 21) |
| --- | --- |
| **Gender (Women: Men), (n%: n%)** | 12: 9 (57%: 43%) |
| **Age (median (min – max) years)** | 69 (46 - 91) |
| *<= 50 years old,* n (%) | *2 (10%)* |
| *>= 80 years old,* n (%) | *4 (19%)* |
| **Ethnicity** |  |
| *White* | *19 (90%)* |
| *South Asian* | *2 (10%)* |
| **Country of Residence** |  |
| *UK, n (%)* | *16 (76%)* |
| *United States of America, n (%)* | *5 (24%)* |
| **Education** |  |
| *Compulsory Education (e.g., GSCE/O-Levels, equivalent)* | *1 (5%)* |
| *Further education (e.g., A-Levels, BTEC, equivalent)* | *2 (10%)* |
| *Higher education below degree (e.g., HNC, HND, diploma)* | *5 (24%)* |
| *Undergraduate degree* | *6 (29%)* |
| *Postgraduate degree* | *7 (33%)* |
| **Socioeconomic status area** |  |
| *Most deprived (1-3 IMD/equivalent)* | *2 (10%)* |
| *Moderately deprived (4-6 IMD/equivalent)* | *6 (29%)* |
| *Least deprived (7-10 IMD/equivalent)* | *13 (62%)* |
| **Rurality** |  |
| *Rural* | *4 19%)* |
| *Urban* | *17 (81%)* |
| **In employment, n (%)** | 4 (19%) |
| **Sexuality** |  |
| *Straight, n (%)* | *14 (67%)* |
| *Gay, n (%)* | *6 (29%)* |
| *Lesbian, n (%)* | *1 (5%)* |

### **Appendix 4.** Demographics of experts

| Demographics | Experts (N = 9) |
| --- | --- |
| **Professional Background** |  |
| *Both technical and clinical* | *2 (22%)* |
| *Clinical* | *6 (67%)* |
| *Technical* | *1 (11%)* |
| **Gait analysis expertise** | *8 (89%)* |
| **Real-world mobility expertise** | *9 (100%)* |

### **Appendix 5.** Proportional bothersomeness scores with stratification of time since diagnosis

|  | **All participants (N=72)** | | | | **Participants diagnosed ≤ 3 years (n=25)** | | | | **Participants diagnosed 3-10 years (n=32)** | | | | **Participants diagnosed >10 years (n=11)** | | | |
| --- | --- | --- | --- | --- | --- | --- | --- | --- | --- | --- | --- | --- | --- | --- | --- | --- |
| **Mobility Symptom (Percentage of People Experiencing Symptom)** | Number of Participants Experiencing the Symptom (n) | Percentage of Participants Experiencing the Symptom (%) | Average Bothersomeness Rating of Symptom (0-10) | Proportional Bothersome Score (0-10) | Number of Participants Experiencing the Symptom (n) | Percentage of Participants Experiencing the Symptom (%) | Average Bothersomeness Rating of Symptom (0-10) | Proportional Bothersome Score (0-10) | Number of Participants Experiencing the Symptom (n) | Percentage of Participants Experiencing the Symptom (%) | Average Bothersomeness Rating of Symptom (0-10) | Proportional Bothersome Score (0-10) | Number of Participants Experiencing the Symptom (n) | Percentage of Participants Experiencing the Symptom (%) | Average Bothersomeness Rating of Symptom (0-10) | Proportional Bothersome Score (0-10) |
| Shuffling | 62 | 86 | 5.80 | 5.00 | 21 | 84 | 6.06 | 5.09 | 28 | 88 | 5.61 | 4.91 | 9 | 82 | 4.44 | 3.64 |
| Bradykinesia | 55 | 76 | 5.57 | 4.26 | 17 | 68 | 5.37 | 3.65 | 26 | 81 | 5.21 | 4.23 | 9 | 82 | 5.88 | 4.81 |
| Balance issues | 53 | 74 | 5.46 | 4.02 | 18 | 72 | 5.23 | 3.77 | 24 | 75 | 5.17 | 3.88 | 9 | 82 | 5.33 | 4.36 |
| Fatigue and Weakness | 41 | 57 | 6.78 | 3.86 | 12 | 48 | 5.80 | 2.78 | 17 | 53 | 7.13 | 3.79 | 10 | 91 | 6.56 | 5.96 |
| Cramps and Dystonia | 44 | 61 | 5.66 | 3.46 | 12 | 48 | 5.27 | 2.53 | 21 | 66 | 5.00 | 3.28 | 8 | 73 | 8.00 | 5.82 |
| Pains | 41 | 57 | 5.63 | 3.21 | 10 | 40 | 5.09 | 2.04 | 19 | 59 | 5.53 | 3.29 | 9 | 82 | 5.50 | 4.50 |
| Freezing of Gait | 24 | 33 | 6.55 | 2.18 | 6 | 24 | 6.00 | 1.44 | 14 | 44 | 7.00 | 3.06 | 2 | 18 | 2.00 | 0.36 |
| Stooped Posture | 23 | 32 | 5.00 | 1.60 | 8 | 32 | 5.00 | 1.60 | 7 | 22 | 3.33 | 0.73 | 7 | 64 | 6.67 | 4.24 |
| Dyskinesia | 18 | 25 | 4.94 | 1.24 | 3 | 12 | 3.33 | 0.40 | 10 | 31 | 5.89 | 1.84 | 5 | 45 | 5.20 | 2.36 |
| Rigidity in Gait | 12 | 17 | 6.00 | 1.00 | 4 | 16 | 6.50 | 1.04 | 7 | 22 | 5.25 | 1.15 | 1 | 9 | 8.00 | 0.73 |

*Note: 4 participants did not report time since diagnosis, hence there is a discrepancy the number of participants stratified by time since diagnosis.

### **Appendix 6.** Proportional impact for each mobility symptom

|  | **All participants (N=72)** | | | | **Participants diagnosed ≤ 3 years (n=25)** | | | | **Participants diagnosed 3-10 years (n=32)** | | | | **Participants diagnosed >10 years (n=11)** | | | |
| --- | --- | --- | --- | --- | --- | --- | --- | --- | --- | --- | --- | --- | --- | --- | --- | --- |
| **Mobility Symptom (Percentage of People Experiencing Symptom)** | Number of Participants Experiencing the Symptom (n) | Percentage of Participants Experiencing the Symptom (%) | Number of Participants Experiencing Impacts Related to the Symptom (n) | Proportional Impact (%) | Number of Participants Experiencing the Symptom (n) | Percentage of Participants Experiencing the Symptom (%) | Number of Participants Experiencing Impacts Related to the Symptom (n) | Proportional Impact (%) | Number of Participants Experiencing the Symptom (n) | Percentage of Participants Experiencing the Symptom (%) | Number of Participants Experiencing Impacts Related to the Symptom (n) | Proportional Impact (%) | Number of Participants Experiencing the Symptom (n) | Percentage of Participants Experiencing the Symptom (%) | Number of Participants Experiencing Impacts Related to the Symptom (n) | Proportional Impact (%) |
| Balance issues | 53 | 74 | 45 | 85 | 18 | 72 | 14 | 78 | 24 | 75 | 20 | 83 | 9 | 82 | 8 | 89 |
| Bradykinesia | 55 | 76 | 35 | 64 | 17 | 68 | 10 | 59 | 26 | 81 | 15 | 58 | 9 | 82 | 8 | 89 |
| Fatigue and Weakness | 41 | 57 | 23 | 56 | 12 | 48 | 8 | 67 | 17 | 53 | 7 | 41 | 10 | 91 | 6 | 60 |
| Shuffling | 62 | 86 | 32 | 52 | 21 | 84 | 12 | 57 | 28 | 88 | 16 | 57 | 9 | 82 | 2 | 22 |
| Dyskinesia | 18 | 25 | 9 | 50 | 3 | 12 | 1 | 33 | 10 | 31 | 6 | 60 | 5 | 45 | 2 | 40 |
| Freezing of Gait | 24 | 33 | 10 | 42 | 6 | 24 | 3 | 50 | 14 | 44 | 6 | 43 | 2 | 18 | 0 | 0 |
| Cramps and Dystonia | 44 | 61 | 13 | 30 | 12 | 48 | 4 | 33 | 21 | 66 | 5 | 24 | 8 | 73 | 3 | 38 |
| Pains | 41 | 57 | 12 | 29 | 10 | 40 | 4 | 40 | 19 | 59 | 6 | 32 | 9 | 82 | 2 | 22 |
| Rigidity in Gait | 12 | 17 | 3 | 25 | 4 | 16 | 1 | 25 | 7 | 22 | 2 | 29 | 1 | 9 | 0 | 0 |
| Stooped Posture | 23 | 32 | 2 | 9 | 8 | 32 | 2 | 25 | 7 | 22 | 0 | 0 | 7 | 64 | 0 | 0 |

*Note: 4 participants did not report time since diagnosis, hence there is a discrepancy the number of participants stratified by time since diagnosis.

### **Appendix 7.** Proportional bothersomeness scores and impact for each mobility symptom stratified by disease stage

**A**

**B**

A: Proportional bothersomeness of each mobility symptom; B: Proportional impact of each mobility symptom

### **Appendix 8.** Overall rankings given to the relevance of DMOs to symptoms

|  | Quality of mobility | | | | | Quantity of mobility | | | | |
| --- | --- | --- | --- | --- | --- | --- | --- | --- | --- | --- |
|  | Walking Speed | Stride Length | Cadence | Turn Duration | Step to Step Variability | Walking Bout Duration | Number of Walking Bouts | Bout to Bout Variability | Step Count | Amount of Activity |
| Balance Issues | 5 | 4 | 3 | 9 | 5 |  |  |  |  |  |
| Shuffling | 7 | 10 |  | 7 | 8 | 2 |  | 7 | 1 | 3 |
| Arm Swing | 4 | 8 | 5 | 10 |  | 5 |  |  |  | 4 |
| Asymmetry of  Motor Symptoms | 9 | 4 | 2 | 2 | 1 | 6 | 6 |  | 5 | 5 |
| Bradykinesia | 8 | 9 | 8 | 8 | 5 |  | 1 |  | 2 | 2 |
| Dyskinesia | 5 | 5 | 3 | 10 | 5 | 2 | 1 | 4 | 2 | 2 |
| Pains | 2 |  |  | 1 |  | 8 | 10 | 9 | 10 | 1 |
| Movement Initiation | 10 | 5 | 6 | 9 | 6 | 1 | 1 | 3 | 4 |  |
| Cramps and Dystonia | 9 | 4 | 2 |  | 1 | 8 | 8 |  | 7 | 6 |
| Freezing of Gait |  | 4 | 8 |  |  |  |  |  |  |  |
| Rigidity in Gait | 10 | 8 | 7 | 11 |  |  |  |  |  |  |
| Stooped Posture | 7 | 8 | 6 | 8 | 3 | 2 |  |  | 9 |  |
| Fatigue and Weakness | 3 |  |  |  | 1 |  |  |  |  |  |

*Note: A higher number denotes greater relevance*

### References

1.	Manta C, Patrick-Lake B, Goldsack Jennifer C. Digital Measures That Matter to Patients: A Framework to Guide the Selection and Development of Digital Measures of Health. Digital Biomarkers. 2020;4(3):69-77.

2.	Pashmdarfard M, Azad A. Assessment tools to evaluate Activities of Daily Living (ADL) and Instrumental Activities of Daily Living (IADL) in older adults: A systematic review. Med J Islam Repub Iran. 2020;34:33.

3.	Taylor JK, Buchan IE, van der Veer SN. Assessing life-space mobility for a more holistic view on wellbeing in geriatric research and clinical practice. Aging Clinical and Experimental Research. 2019;31(4):439-45.

4.	Allen NE, Schwarzel AK, Canning CG. Recurrent Falls in Parkinson’s Disease: A Systematic Review. Parkinson's Disease. 2013;2013(1):906274.

5.	Park CL, Kubzansky LD, Chafouleas SM, Davidson RJ, Keltner D, Parsafar P, et al. Emotional Well-Being: What It Is and Why It Matters. Affective Science. 2023;4(1):10-20.

6.	Viceconti M, Tome M, Dartee W, Knezevic I, Hernandez Penna S, Mazzà C, et al. On the use of wearable sensors as mobility biomarkers in the marketing authorization of new drugs: A regulatory perspective. Frontiers in Medicine. 2022;9.

7.	Park JH, Kang YJ, Horak FB. What Is Wrong with Balance in Parkinson's Disease? J Mov Disord. 2015;8(3):109-14.

8.	Sveinbjornsdottir S. The clinical symptoms of Parkinson's disease. Journal of Neurochemistry. 2016;139(S1):318-24.

9.	Ford B. Pain in Parkinson's disease. Movement Disorders. 2010;25(S1):S98-S103.

10.	Nutt JG, Bloem BR, Giladi N, Hallett M, Horak FB, Nieuwboer A. Freezing of gait: moving forward on a mysterious clinical phenomenon. The Lancet Neurology. 2011;10(8):734-44.

11.	Navarro-López V, Fernández-Vázquez D, Molina-Rueda F, Cuesta-Gómez A, García-Prados P, del-Valle-Gratacós M, et al. Arm-swing kinematics in Parkinson's disease: A systematic review and meta-analysis. Gait & Posture. 2022;98:85-95.

12.	Armstrong MJ, Okun MS. Diagnosis and Treatment of Parkinson Disease: A Review. JAMA. 2020;323(6):548-60.

13.	Delval A, Tard C, Defebvre L. Why we should study gait initiation in Parkinson's disease. Neurophysiologie Clinique/Clinical Neurophysiology. 2014;44(1):69-76.

14.	Bologna M, Espay AJ, Fasano A, Paparella G, Hallett M, Berardelli A. Redefining Bradykinesia. Movement Disorders. 2023;38(4):551-7.

15.	Giladi N, Shabtai H, Rozenberg E, Shabtai E. Gait festination in Parkinson's disease. Parkinsonism & Related Disorders. 2001;7(2):135-8.

16.	Morris ME, Huxham F, McGinley J, Dodd K, Iansek R. The biomechanics and motor control of gait in Parkinson disease. Clinical Biomechanics. 2001;16(6):459-70.

17.	Verbaan D, Marinus J, Visser M, van Rooden SM, Stiggelbout AM, van Hilten JJ. Patient-reported autonomic symptoms in Parkinson disease. Neurology. 2007;69(4):333-41.

18.	Williams-Gray CH, Worth PF. Parkinson's disease. Medicine. 2016;44(9):542-6.

19.	Peralta CM, Frauscher B, Seppi K, Wolf E, Wenning GK, Högl B, et al. Restless legs syndrome in Parkinson's disease. Movement Disorders. 2009;24(14):2076-80.

20.	Lumsdon J, Wilson C, Alcock L, Becker C, Benvenuti F, Bonci T, et al. Cocreating the Visualization of Digital Mobility Outcomes: Delphi-Type Process With Patients. JMIR Form Res. 2025;9:e68782.

21.	Claudia M, Lisa A, Kamiar A, Clemens B, Stefano B, Tecla B, et al. Technical validation of real-world monitoring of gait: a multicentric observational study. BMJ Open. 2021;11(12):e050785.

22.	Rochester L, Mazzà C, Mueller A, Caulfield B, McCarthy M, Becker C, et al. A Roadmap to Inform Development, Validation and Approval of Digital Mobility Outcomes: The Mobilise-D Approach. Digital Biomarkers. 2020;4(Suppl. 1):13-27.
